# Determinants of postnatal care non-utilization among women in Nigeria

**DOI:** 10.1186/s13104-015-1823-3

**Published:** 2016-01-11

**Authors:** Oluwaseyi Dolapo Somefun, Latifat Ibisomi

**Affiliations:** Demography and Population Studies, Faculty of Humanities, University of the Witwatersrand, 2000 Johannesburg, South Africa; School of Public Health and Epidemiology, University of the Witwatersrand, Johannesburg, South Africa

**Keywords:** Antenatal care, Distance, DHS, Maternal mortality, Nigeria, Postnatal care, Place of delivery

## Abstract

**Background:**

Although, there are several programs in place in Nigeria to ensure maternal and child health, maternal and neonatal mortality rates remain high with maternal mortality rates being 576/100,000 and neonatal mortality rates at 37/1000 live births (NDHS, 2013). While there are many studies on the utilization of maternal health services such as antenatal care and skilled delivery at birth, studies on postnatal care are limited. Therefore, the aim of this study is to examine the factors associated with the non-utilization of postnatal care among mothers in Nigeria using the Nigeria Demographic and Health Survey (NDHS) 2013.

**Methods:**

For analysis, the postnatal care uptake for 19,418 children born in the 5 years preceding the survey was considered. The dependent variable was a composite variable derived from a list of questions on postnatal care. A multinomial logistic regression model was applied to examine the adjusted and unadjusted determinants of non-utilization of postnatal care.

**Results:**

Results from this study showed that 63 % of the mothers of the 19,418 children did not utilize postnatal care services in the period examined. About 42 % of the study population between 25 and 34 years did not utilize postnatal care and 61 % of the women who did not utilize postnatal care had no education. Results from multinomial logistic regression show that antenatal care use, distance, education, place of delivery, region and wealth status are significantly associated with the non-utilization of postnatal care services.

**Conclusions:**

This study revealed the low uptake of postnatal care service in Nigeria. To increase mothers’ utilization of postnatal care services and improve maternal and child health in Nigeria, interventions should be targeted at women in remote areas who don’t have access to services and developing mobile clinics. In addition, it is crucial that steps should be taken on educating women. This would have a significant influence on their perceptions about the use of postnatal care services in Nigeria.

## Background

Maternal health is a key health challenge globally. The unacceptably high levels of maternal mortality are a common subject in global health and development discussions. Although some countries have made remarkable progress, half of the maternal deaths in the world still take place in Sub-Saharan Africa where little or no progress has been made. For instance, the maternal mortality rate (MMR) in Eastern Asia decreased by 69 % followed by Northern Africa (66 %), Southern Asia (64 %) compared to sub-Saharan Africa (41 %), [1]. Also, in 2010 the MMR was estimated to be 470 per 100,000 in Zimbabwe, 400 in Burkina Faso, 380 in Ghana and 560 in Nigeria [2]. There is no single simple, straightforward intervention that will significantly decrease maternal mortality; however, different studies have documented the importance of a strong health system, skilled delivery attendants, and appropriate postnatal care uptake [3–5].

According to the NDHS (2013), more than half (56 percent) of women did not receive any postnatal care [6]. The survival and well-being of a woman and her new-born depends substantially on the care received during pregnancy, delivery and the postnatal period as a large number of maternal and neonatal deaths occur during the first 24 h after delivery [7]. Neonatal mortality in the first week of life accounts for approximately 75 percent of all neonatal death and majority of these deaths occur in developing countries. Most of these new-born deaths are due to sepsis, asphyxia and problems associated with low birth weight [8, 9]. Studies show that deaths within the first week of life account for almost 40 % of all deaths among children under the age of five [8, 9]. Also, about 700 babies die (around 30 every hour) on a daily basis in sub-Saharan Africa which has the highest number of new-born deaths in Africa, and the second highest in the world [10].

A large number of women in the sub-Saharan region do not have access to healthcare during the early postnatal period which puts them at risk of diseases and mortality [11]. The harsh reality is that about 4 million infants do not live through the immediate postnatal period, and a large number of them are disabled due to pregnancies and births that are poorly monitored or handled [12]. Ensuring a safe motherhood and a healthy childhood remains a major challenge in sub-Saharan Africa and Nigeria is no exception. The rate of maternal mortality (560 per 100,000 live births) [2] and perinatal mortality (78 per 1000 pregnancies) in Nigeria is particularly very high [13]. A recent report by the Federal Ministry of Health [14] showed that nearly a quarter of a million babies die annually in Nigeria and there has been no significant reduction in the average neonatal mortality rate. Many of the maternal deaths occur at home and are therefore unaccounted for in the official statistics [15]. Given that the country’s population is the largest in Africa, it has a significant influence on the rest of sub-Saharan Africa’s performance and contributes disproportionately to global childhood mortality crisis.

Postnatal care is an aspect of child survival that has received limited attention in Nigeria. This situation is tragic, especially as most of these babies die from preventable causes, such as: intrapartum- related injury, infections, and prematurity [14]. Further, there is limited research on factors associated with non-utilization of postnatal care in Nigeria. The few studies that have been carried out in Nigeria have been at local geographic regions [16] or have looked at adolescents [17]. Given the importance of postnatal care for the wellbeing and survival of mother and child, and the documented poor uptake of postnatal care, a study at national level is important as it may help in directing policies that address the issue of postnatal care non utilization at national level. In addition, the few studies that exist on postnatal care in Nigeria have concentrated on women who make use of these services with little attention on factors that are associated with women, who do not utilize postnatal care. This study has the potential to identify the factors or determinants of postnatal care non-utilization. Identification of these factors will give clues on how the issues can be tackled.

## Methods

This is a secondary data analysis of a population-based cross-sectional data of the 2013 Nigerian Demographic and Health Survey (NDHS). The child recode dataset was used for this study. This dataset has one record for every child born in the 5 years preceding the survey for interviewed women. It contains the information related to the child’s pregnancy, delivery, postnatal care and immunization among others. The data for the mother of each of these children are included. For this study, only the last child of the women were used to avoid mix-ups in the recalling and reporting of mothers experiences, especially when they have had more than one birth in the last 5 years. Also, women who had experienced child mortality within 42 days were dropped. This amounted to 19,418 live births born to 19,418 women (between 15 and 49 years) in the 5 years preceding the 2013 Nigeria Demographic and Health Survey.

### Outcome variable

The outcome of this study was non-utilization of postnatal care services. The variable was constructed using the WHO definition of postnatal care, which takes into account attendance of PNC services, checked by trained health personnel and timing of care (within 42 days of birth). It was thus derived from the following questions:

1. After discharge/delivery at home, anyone checked respondent?
2. Who checked respondent’s health after discharge?
3. How long after discharge/delivery at home was respondent’s health checked?

Qualified health care providers are country specific and variations exist. For the purpose of this study, which draws reference from other studies, qualified health care providers include; doctors, nurses, midwives, community extension health worker, community or village health workers and auxiliary midwives, while unqualified health care providers include traditional birth attendants, and ‘others’. In Nigeria, Community Health Officers (CHOs) and Community Extension Health Workers (CHEW) are trained at the schools of health technology which is why we have included them in the analysis as qualified health care providers. With regards to timing, it is recommended that the mother and baby be assessed within 1 h of birth and again before discharge if the mother is in a facility, and also within first 42 days. For births that occur at home, first visits should target the crucial 24 h after birth, and a further visit within first 42 days. Thus, the categorization of PNC as use and non-use in this study complies with the highest level of PNC (appropriate care). Mothers were considered to have made adequate utilization of PNC services if she and are baby were checked by qualified healthcare personnel within 42 days of child birth. Hence, PNC non-utilization was categorized in this study as “0” if respondent was attended to by a qualified health worker within 42 days; “1” if respondent was not attended to by qualified healthcare personnel within 42 days of birth; and “2” if respondent had one or two of the three essential components of post-natal care (i.e. any one or two of post-natal visit, attendance by skilled health care worker and within 42 days). These are labelled non-utilization, some level of care and appropriate care, respectively.

Given the poor uptake of PNC, a study at national level is important as it may help in directing policies that address the issue of PNC non utilization at national level. A geographically broad, quantitative assessment of women’s reasons for non-use can help shed light on general patterns and trends regarding the relative importance of these reasons.

### Independent variables

The independent variables used in this study include demographic, social and economic variables and were selected based on their documented association with postnatal care utilization [17–20].

These are: Age of mother at last birth, antenatal care use, birth order, birth size, child sex, distance to health facility, pregnancy wantedness, education, marital status, occupation, place of delivery, place of residence, region, religion and household wealth status. A visit to a qualified health care provider for ANC irrespective of timing and frequency was used as a proxy for antenatal care. See Table 3 in Appendix for a summary of all variables used in the study.

### Statistical analysis

Three levels of analysis were employed in this paper. These were bivariate descriptive, unadjusted and adjusted multinomial logistic modelling. At the bivariate descriptive level, the percentage distribution of study sample was presented by the various selected characteristics of mothers and children. Unadjusted and adjusted multinomial logistic regression were then employed to examine the independent and net relationship between all the independent variables and the outcome variable. A *p* value <0.05 was considered statistically significant.

### Ethical consideration

The Nigerian DHS can be downloaded from the website and is free to use by researchers for further analysis. In order to access the data from DHS MEASURE a written request was submitted to the DHS MACRO and permission was granted to use the data for this survey.

### Limitations

This study made use of cross-sectional data. As such, the study was unable to conclusively determine the temporal relationship between the independent variables and dependent variable rather, associations were examined. Also, the 2013 Nigeria Demographic and Health Survey data was collected retrospectively. This may be associated with recall bias given that the events took place 5 years following the survey. For instance, women may forget or may not accurately recall during the interview the number of postnatal care visits attended. In addition, the current DHS data for women in Nigeria does not ask questions on PNC non-use, hence results may not reveal up-to-date situation of PNC non-use.

## Results

Table 1 presents the weighted profile of women in the analysis sample by their non-utilization of postnatal care. Results show that 63 % of the women did not utilize postnatal care within 42 days after delivery and only 4 % of the women utilized the appropriate care according the WHO guidelines. The remaining 33 % of the women had some level of care which means that they had one or two of the three essential components of postnatal care. Among the postnatal care non-users, majority of them were aged 25–34 at birth of the child, Muslims, live in rural areas, married, had no formal education, delivered in non-health facility or were poor.

**Table 1:** Percentage distribution of women by non-utilization of postnatal care in Nigeria

Characteristics	Total N = 19,418	No PNC N = 12,156	Some level of PNC N = 6417	Appropriate PNC N = 845
Mothers age at child birth				
15–24	37.06	39.14	32.87	37.62
25–34	44.35	41.98	49.25	46.96
35+	18.41	18.88	17.87	15.41
Pregnancy wantedness				
Then	90.26	92.88	85.93	83.93
Later	7.59	5.57	10.84	13.18
No more	2.14	1.55	3.23	2.88
Education				
No education	47.85	61.27	24.17	26.57
Primary	18.92	17.17	21.95	22.19
Secondary and higher	33.23	21.56	53.88	51.24
Marital status				
Never married	2.11	1.57	2.82	4.85
Currently married	94.88	96.85	93.44	91.39
Formerly married	3.01	2.59	3.74	3.75
Occupation				
Not working	29.55	32.94	23.25	26.61
Formal employment	8.78	5.90	14.58	8.02
Sales	39.73	38.31	41.90	44.38
Agricultural employment	10.17	10.95	8.73	9.37
Other	11.77	11.90	11.54	11.61
Religion				
Christian	37.82	27.62	56.26	50.56
Islam	61.17	71.07	43.19	49.20
Other	1.01	1.30	0.54	0.24
Wealth status				
Poor	45.17	57.56	23.70	22.79
Middle	19.06	18.85	19.04	22.26
Rich	35.77	23.59	57.36	54.95
Region				
South west	14.64	8.37	25.91	23.04
North central	14.12	14.60	13.47	12.03
North east	16.76	18.03	14.50	14.92
North west	36.33	45.43	19.82	25.40
South east	8.36	6.08	12.87	8.35
South south	9.78	7.49	13.42	16.24
Place of residence				
Urban	35.73	26.51	52.02	50.11
Rural	64.27	73.49	47.98	49.89
Birth order				
1–2	34.04	30.88	39.44	40.31
3–4	28.59	27.80	30.04	29.33
5+	37.37	41.32	30.52	30.36
Birth size				
Larger than average	44.42	43.83	45.63	43.94
Average	41.03	39.40	43.71	44.83
Smaller than average	14.56	16.76	10.66	11.22
Child sex				
Male	49.96	49.70	50.31	51.30
Female	50.04	50.30	49.69	48.70
ANC use				
No ANC	38.70	50.97	17.41	17.47
Received ANC	61.30	49.03	82.59	82.53
Distance				
Big problem	31.78	38.65	19.64	21.22
Not a big problem	68.22	61.35	80.36	78.78
Place of delivery				
Health facility	37.54	26.47	62.85	11.60
Non-health facility	62.46	73.53	37.15	88.40

The results of the bivariate model are presented in the second panel of Table 2. The findings show that, pregnancy wantedness, mothers education, marital status, occupation, place of residence, region, religion wealth status, child’s birth order, child’s birth size, mothers use of ANC services, distance to health facility place of delivery are significant factors affecting postnatal care non- utilization.

**Table 2:** Unadjusted and adjusted relative risk ratios of association between selected women and children characteristics and postnatal care non-utilization in Nigeria

Characteristics	Unadjusted		Adjusted	
	Non-utilization vs appropriate PNC	Some level of care vs appropriate PNC	Non-utilization vs appropriate PNC	Some level of care vs appropriate PNC
	RR	RR	RR	RR
Mothers age at child birth				
15–24	1.00	1.00	1.00	1.00
25–34	0.88	1.18*	0.76*	0.86
35+	1.12	1.29*	0.78	0.89
Pregnancy wantedness				
Then	1.00	1.00	1.00	1.00
Later	0.41*	0.79*	0.78*	0.94
No more	0.50*	0.93	0.73	0.87
Education				
No education	1.00	1.00	1.00	1.00
Primary	0.31*	0.99	0.52*	0.84
Secondary and higher	0.21*	1.17*	0.38*	0.65*
Marital status				
Never married	1.00	1.00	1.00	1.00
Currently married	2.58*	1.49*	1.02	1.08
Formerly married	1.86*	1.37	0.87	0.96
Occupation				
Not working	1.00	1.00	1.00	1.00
Formal employment	0.53*	1.85*	1.15	1.31*
Sales	0.70*	1.05	0.81	1.01
Agricultural employment	0.76*	0.90	1.00	0.90
Other	0.84	1.08	1.02	1.19
Religion				
Christian	1.00	1.00	1.00	1.00
Islam	2.39*	0.83*	1.16	0.97
Other	9.49*	2.31	4.17*	2.81
Region				
South west	1.00	1.00	1.00	1.00
North central	2.10*	0.86	1.81*	1.25
North east	2.68*	0.80*	1.66*	1.63*
North west	5.35*	0.66*	3.37*	1.54*
South east	2.04*	1.41*	1.45*	1.09
South south	1.30*	0.74*	1.93*	1.42*
Wealth status				
Poor	1.00	1.00	1.00	1.00
Middle	0.33*	0.86	0.57*	0.80
Rich	0.21*	1.13	0.38*	0.66*
Place of residence				
Urban	1.00	1.00	1.00	1.00
Rural	2.06*	0.78*	1.05	1.11
Birth order				
1–2	1.00	1.00	1.00	1.00
3–4	1.22*	1.03	1.37*	1.20*
5+	1.61*	0.98	1.53*	1.30*
Birth size				
Larger than average	1.00	1.00	1.00	1.00
Average	1.03	0.97	1.01	1.01
Smaller than average	1.54*	0.94	1.24	1.03
Child sex				
Male	1.00	1.00	1.00	1.00
Female	1.06	1.04	1.05	1.07
ANC use				
No ANC	1.00	1.00	1.00	1.00
Received ANC	0.21*	0.95	0.25*	0.46*
Distance				
Big problem	1.00	1.00	1.00	1.00
Not a big problem	0.44*	0.99	0.62*	0.83*
Place of delivery				
Health facility	1.00	1.00	1.00	1.00
Non-health facility	0.31*	0.07*	0.05*	0.03*

* P < 0.05

The second panel of Table 2 shows the adjusted coefficients of the association between selected children and mothers characteristics with postnatal care non-utilization. In this model, age of mother at birth of child, pregnancy wantedness, mothers education, region, religion, wealth status, child’s birth order, distance to health facility, place of delivery, and were statistically significant while marital status, occupation, place of residence and birth size lost the significance that they had at the bivariate level. ANC use,

The relative risk of not utilizing postnatal care for the mothers aged 25+ at birth of child (RRR = 0.76, CI = 0.62–0.94) was lower than that or mothers aged 15–24 and this association was statistically significant. The relative risk of postnatal care non-utilization for women who said they wanted pregnancy later (RRR = 0.78, CI 0.60–1.01) was seen to be lower compared to women who wanted the pregnancy. Mothers with high parity (5 and above) were 1.53 times more likely to not use postnatal care (RRR = 1.53, CI 1.18–1.99). A negative association was found between educational attainment and PNC non-utilization as the non-utilization of postnatal care decreased with an increase in the educational level. With regards to religion, the relative risk of not utilizing postnatal care was higher for women who practiced “other” religion (RRR = 4.17, CI 0.99–17.44) forms of religion. The odds of not utilizing postnatal care services decreased as the household wealth index increased. Women who belonged to the middle category (RRR = 0.57, CI 0.46–0.72) had lower odds of not utilizing postnatal care and women in the rich category (RRR = 0.38, CI 0.30–0.49) also had lower odds of not utilizing postnatal care. The odds of not utilizing appropriate postnatal care were significantly higher in all the other regions compared to the South West.

The relative risk of not using postnatal care was significantly lower for women who received antenatal care (RRR = 0.25, CI 0.21–0.31) compared to women who did not receive. With respect to distance to health facility, women who stated that distance was not a problem (RRR = 0.62, CI 0.51–0.75) had significantly lower odds of not using postnatal care services. Mothers of children who were delivered in a non-health care facility have lower odds (RRR = 0.05, CI 0.04–0.06) of not utilizing postnatal care compared to mothers who delivered in a health care facility.

## Discussion

The objective of this study was to identify factors that are significantly associated with the non-use of postnatal care services (a significant and often neglected part of maternal and child health care) in Nigeria. The findings show that the uptake of appropriate postnatal care utilization is very low in Nigeria, as previously documented in the country [16, 18]. About 63 % of the women who had births in the 5 years preceding the survey did not utilize postnatal care. This could be due to a high number (63 %) of home births by the mothers in Nigeria [6]. Our results differ from 2013, NDHS report which reported that 58 % of women did not receive any postnatal care within 41 days of delivery. This may be due to the categorization of PNC in this study which defined skilled personnel as: doctors, nurses, midwives, community or village health workers, auxiliary midwives and community health officers and also because we did not consider women who utilized PNC services after 42 days. Further, we restricted analysis to last births of the women and for only children that survived beyond 41 days of life.

This study identified the following factors as having an important influence on non-utilization of postnatal care services in Nigeria. Most of these findings are consistent with previous studies [21–24]. Age of mother as at birth of child, pregnancy wantedness, education, wealth status, mothers use of ANC services, distance to health facility and place of delivery were associated with lower odds or not utilizing PNC services while religion, region and birth order were associated with increased odds of not utilizing PNC services. The identification of these factors are important in developing public health policies and interventions on increasing the utilization of postnatal care services in Nigeria and elsewhere.

Education emerged as a significant determinant of PNC non-utilization. It has been argued that better educated women understand the importance of postnatal care and are more likely to know where to get it [25] than less educated women. This finding is in line with those of many other studies. Education is likely to empower an individual to gain access to health promotion message, information to obtain services and importance of the available services. Likewise, educated individuals are likely to be able to process the health message. Elsewhere, it has been documented that educated women are more aware of health problems, have more knowledge on the availability of health care services, and use this information more effectively to maintain or achieve good health [26]. In addition, educated women may have higher socioeconomic status which may translate to having higher autonomy in their households and their ability for making independent decisions on their health utilization [25, 27, 28]. It was found that smaller than average-sized babies were more likely not to utilize PNC services. This is an unexpected finding as we expect that mothers of children with low birth weight may utilize PNC services more due to the observed fragility of small babies. This study shows that the household wealth status of mothers is significantly associated with their utilization of postnatal care services. This finding is in line with various other studies that point out the significant economic inequality in healthcare service utilization [20, 29–31]. It has been hypothesized that poor households may not have the resources for healthcare expenses, because their priority is to meet their basic daily needs, whereas wealthier households can spend a higher proportion of their earnings on healthcare [32, 33].

Mothers of children who reported that distance to health facility was not a big problem had lower odds of not utilizing PNC services. This finding is consistent with those of other studies conducted in Africa and other developing countries, which confirm that physical proximity [24] and geographical distance [23] play an important role in the utilization of maternal health services. The lower utilization of PNC services among mothers who stated that distance was a problem could be linked to their socio-economic status. The majority of the women in the rural areas may not be able to afford the cost of transport to these health facilities. However, improved electricity, transportation, water, and sanitation services are, on average, more widely available in urban areas, and could enhance a mother’s utilization of PNC services [21]. In addition, greater awareness of health promotion programs and access to services among urban women could have a positive impact on the utilization of healthcare services [22]. This study also showed that mothers who received ANC from a skilled provider had lower odds of not utilizing PNC services. This is consistent with other findings [16, 17, 34–36].

The analysis showed a highly significant association between the place of delivery and the utilization of postnatal care services where children delivered in a non-health care facility had lower odds of not utilizing PNC services. This finding differs from that of the study done in Indonesia and Nepal which found that infants delivered outside a health care facility were significantly less likely to utilize postnatal care services [19, 37]. Our results may probably be because mothers who deliver at the health facility may feel quite confident about their health and the health of their new-born and may not see the need to return for check-ups while those that did not deliver in health facility would like the health of the child to be checked and hence, are more likely to utilize PNC services.

The results from this study suggest that public health policies and programmes should aim to improve postnatal care utilization through: education and empowerment of women with skills that can assist them make appropriate decision and adequate livelihood. The need to avail maternal services within reasonable distance to households within communities is also crucial. All these can be achieved through community-based outreach programmes that provide reproductive health information and services, and address a wide range of needs including life skills, literacy, vocational training, and livelihood activities.

## Appendix

See Table 3.

**Table 3:** Variable definition and codes of selected variables

Variable code	Variable name	Original variable categories	How it is coded in this study
V008, 011, v222	Mothers age at birth of child	Difference between Continuous variables measuring date of interview date of birth and last birth to interview were generated and divided by 12	15–24 (1) 25–34 (2) 35+ (3)
M10	Pregnancy wantedness	Then Later No More	Then (1) Later (2) No more (3)
V106	Education	No education Primary Secondary Higher	No education (1) Primary (2) Secondary and Higher (3)
V502	Marital status	Never married Currently married Formerly married	Never married (1) Currently married (2) Formerly married (3)
V717	Occupation	Not working Prof; tech, manag Clerical Sales Agric-employee Services Skilled manual Unskilled manual	Not Working (1) Formal employment (2) which included prof. and clerical workers Sales (3) Agricultural employment (4) Other (5) which included services, skilled manual and unskilled manual
V130	Religion	Catholic Other Christian Islam Traditionalist Other	Christian (1) Islam (2) Traditionalist/other (3)
V190	Wealth status	Poorest Poorer Middle Richer Richest	Poor (1) Middle (2) Rich (3)
V101	Region	North Central North East North West South East South South South West	South West (1) North Central (2) North East (3) North West (4) South East (5) South South (6)
V102	Place of residence	Urban Rural	Urban (1) Rural (2)
Bord	Birth order	Continuous variable (1–18)	1–2 (1) 3–4 (2) 5+ (3)
M18	Birth size	Very large Larger than average Average Smaller than average Very small	Larger than average (1) Average (2) Smaller than average (3)
B4	Child sex	Male Female	Male (1) Female (2)
M2a, M2b, M2c	Antenatal care use	No Yes	No (1) Yes (2)
V467d	Distance	Big problem Not a big problem	Big problem (1) Not a big problem (2)
M15	Place of delivery	Respondents home Other home Government hospital Govt. health centre Govt. health post Other public Private hospital/clinic Other private medical	Heath facility (1) Non-health facility (2) where respondents home and other home were categorized as non-health facility

## Abbreviations

ANC: antenatal care
AIDS: ACQUIRED Immunodeficiency Syndrome
CIA: Central Intelligence Agency
CSO: Central Statistics Office
DHS: Demographic and Health Surveys
HIV: human immunodeficiency virus
MMR: maternal mortality ratio
MDG: millennium development goals
NMR: neonatal mortality rate
NDHS: Nigeria Demographic and Health Survey
NPC: Nigerian population commission
OR: odds ratios
PNC: postnatal care
UN: United Nations
UNFPA: United Nations Population Fund
UNICEF: United Nations Children’s Fund
USAID: United States Agency for International Development
WHO: World Health Organization

## References

[1] WHO, Trends in Maternal Mortality: 1990–2010. Estimates developed by WHO, UNICEF, UNFPA and the World Bank. Geneva: WHO. 2012.

[2] WHO. World Health Statistics 2014. Geneva, World Health Organization. 2014.

[3] Singh PK (2014). Factors associated with maternal healthcare services utilization in nine high focus states in India: a multilevel analysis based on 14 385 communities in 292 districts. Health Policy Plan.

[4] Pattinson R (2011). Stillbirths: how can health systems deliver for mothers and babies?. Lancet.

[5] Blank A (2013). “Quality of prenatal and maternal care: bridging the know-do gap”(QUALMAT study): an electronic clinical decision support system for rural Sub-Saharan Africa. BMC Med Inform Decis Mak.

[6] NPC. Nigeria Demographic and Health Survey 2013. Abuja, Nigeria: National Population Commission and ICF Macro Nigeria: National Population Commission (NPC) and ICF Macro 2013.

[7] Lawn JE (2005). 4 million neonatal deaths: when? Where? Why?. The Lancet.

[8] WHO. Newborns: reducing mortality [Internet]. World Health Organisation 2012 [cited August 4 2014]. 2012.

[9] Lawn JE et al., Newborn survival. 2006.

[10] PMNCH. Opportunities for Africa’s newborns: Practical data, policy and programmatic support for newborn care in Africa. The Partnership for Maternal, Newborn and Child Health. 2006.

[11] Lawn JE (2004). Why are 4 million newborn babies dying each year?. The Lancet.

[12] de Bernis L (2003). Skilled attendants for pregnancy, childbirth and postnatal care. Br Med Bull.

[13] Fawole AO (2011). Determinants of perinatal mortality in Nigeria. Int J Gynecol Obstetr.

[14] FMOH. A Directory of Health Facilities in Nigeria 2011. Abuja: Federal Ministry of Health, Nigeria, 2011.

[15] Ibrahim YZS (2014). Temporal analysis of maternal mortality in kano state, northern Nigeria: a six-year review. Am J Public Health Res.

[16] Ugboaja JO (2013). Barriers to postnatal care and exclusive breastfeeding among urbanwomen in southeastern Nigeria. Nigerian Med J.

[17] Rai RK, Singh PK, Singh L (2012). Utilization of maternal health care services among married adolescent women: insights from the Nigeria Demographic and Health Survey, 2008. Women’s Health Issues.

[18] Babalola S, Fatusi A (2009). Determinants of use of maternal health services in Nigeria-looking beyond individual and household factors. BMC Preg Childbirth.

[19] Khanal V (2014). Factors associated with the utilisation of postnatal care services among the mothers of Nepal: analysis of Nepal Demographic and Health Survey 2011. BMC Women’s Health.

[20] Ochako R (2011). Utilization of maternal health services among young women in Kenya: Insights from the Kenya Demographic and Health Survey, 2003. BMC Preg Childbirth.

[21] Fotso J-C (2007). Urban–rural differentials in child malnutrition: trends and socioeconomic correlates in sub-Saharan Africa. Health Place.

[22] Mekonnen Y, Mekonnen A. Utilization of maternal health care services in Ethiopia. 2002.

[23] Rahaman MM (1982). A diarrhea clinic in rural Bangladesh: influence of distance, age, and sex on attendance and diarrheal mortality. Am J Public Health.

[24] Stock R (1983). Distance and the utilization of health facilities in rural Nigeria. Soc Sci Med.

[25] Raghupathy S (1996). Education and the use of maternal health care in Thailand. Soc Sci Med.

[26] Chakraborty N (2003). Determinants of the use of maternal health services in rural Bangladesh. Health Promotion Int.

[27] Cleland JG, Van Ginneken JK (1988). Maternal education and child survival in developing countries: the search for pathways of influence. Soc Sci Med.

[28] Caldwell JC. Education as a factor in mortality decline an examination of Nigerian data. Population Stud. 1979; 395–13.

[29] Elo IT. Utilization of maternal health-care services in Peru: the role of women’s education. Health Trans Rev. 1992; 49–69. 10148665

[30] Fosu GB (1994). Childhood morbidity and health services utilization: cross-national comparisons of user-related factors from DHS data. Soc Sci Med.

[31] Nwogu, E., Utilization of maternity care in Nigeria. Global J Pure Appl Sci. 2009; 15(3–4).

[32] Amin R, Shah NM, Becker S (2010). Socioeconomic factors differentiating maternal and child health-seeking behavior in rural Bangladesh: a cross-sectional analysis. Int J Equity Health.

[33] Singh L, Rai RK, Singh PK (2012). Assessing the utilization of maternal and child health care among married adolescent women: evidence from India. J Biosoc Sci.

[34] Chimankar DA, Sahoo H. Factors influencing the utilization of maternal health care services in Uttarakhand. 2011.

[35] Magoma M (2013). The effectiveness of birth plans in increasing use of skilled care at delivery and postnatal care in rural Tanzania: a cluster randomised trial. Tropical Med Int Health.

[36] Mazambani D (2012). Determinants of maternal healthcare utilization in Zimbabwe. Int J Econ Sci Appl Res.

[37] Titaley CR, Dibley MJ, Roberts CL (2009). Factors associated with non-utilisation of postnatal care services in Indonesia. J Epidemiol Community Health.

